# Validity of humerus fracture classification in the Swedish fracture register

**DOI:** 10.1186/s12891-017-1612-3

**Published:** 2017-06-10

**Authors:** David Wennergren, Stina Stjernström, Michael Möller, Mikael Sundfeldt, Carl Ekholm

**Affiliations:** Department of Orthopaedics, Sahlgrenska University Hospital, SE-413 45 Gothenburg, Mölndal Sweden

**Keywords:** Fracture classification, Validity, Agreement, Accuracy, Humerus fracture, Fracture register

## Abstract

**Background:**

The ability to correctly classify fractures is of importance for choosing the appropriate treatment and for providing appropriate data for research and quality registers. In the Swedish Fracture Register (SFR) fractures of all types are registered by the attending physician, often a junior doctor. For the majority of fractures, a modified AO/OTA classification is used. This study aimed to validate the accuracy of classification of humerus fractures in the SFR and also at providing insight into inherent classification uncertainties.

**Methods:**

One hundred and sixteen humerus fractures (among them 90 proximal) were retrieved by computer randomisation from the SFR and reassessed independently at two occasions, 6 weeks apart, by three senior orthopaedic surgeons blinded to patient information and a consensus “gold standard” classification was established. This was compared with the classifications that had been entered into the register.

**Results:**

The agreement between gold standard classification and original classification in the SFR was kappa = 0.57 for all humerus fractures. For proximal humerus fractures kappa-coefficient for intra-observer agreement was 0.593, 0.599 and 0.752 for the three observers respectively. Taking into account the similarities between certain fracture groups, a modified calculation of agreement was performed. With this modification the intra-observer agreement was 0.910-0.974 and inter-observer agreement was 0.912.

**Conclusion:**

The classification of humerus fractures in the Swedish Fracture Register was just as accurate as in previous studies, i.e. moderate as defined by Landis and Koch. However, when we introduced a modified analysis, that takes into account the similarities between certain fractures, the accuracy was “near perfect”.

## Background

Understanding fracture morphology is an essential step in assessing fractures for appropriate treatment. Regardless of the classification system that is used, inter- and intra-observer agreement have been poor to moderate using plain radiographs [1, 2]. Previous studies of the reliability of fracture classification have been performed using a selection of fractures in a test situation.

Classifying fractures means clustering fracture patterns into different sets. Although the boundaries of the sets may be fairly well defined, the fractures that are going to be classified are part of a continuum. Fractures may display features of two different fracture sets to a varying degree and, to some degree, the assessment by the person working with the system is subjective. Furthermore, in fracture classification, there are no absolutely correct answers but rather degrees of agreement between different assessors.

The Swedish Fracture Register (SFR) is an on-line national fracture registration system in which the individual doctor, who sees the patient at the emergency department and later during treatment, enters data relating to the fracture: fracture date, trauma mechanism, fracture localisation and fracture classification, including multiple fractures [3]. Treatment is registered, primary treatment including non-surgical treatment, as well as secondary procedures. Questionnaires (Eq5D, SMFA) are mailed to the patient 1 year after the injury for follow-up to be compared with the ones completed by the patient at the time of injury regarding his/her pre-injury health (recall technique). In the context of the present study, it should be pointed out that junior doctors who are not specifically trained for this work do the majority of the classification work.

**Fig. 1:**
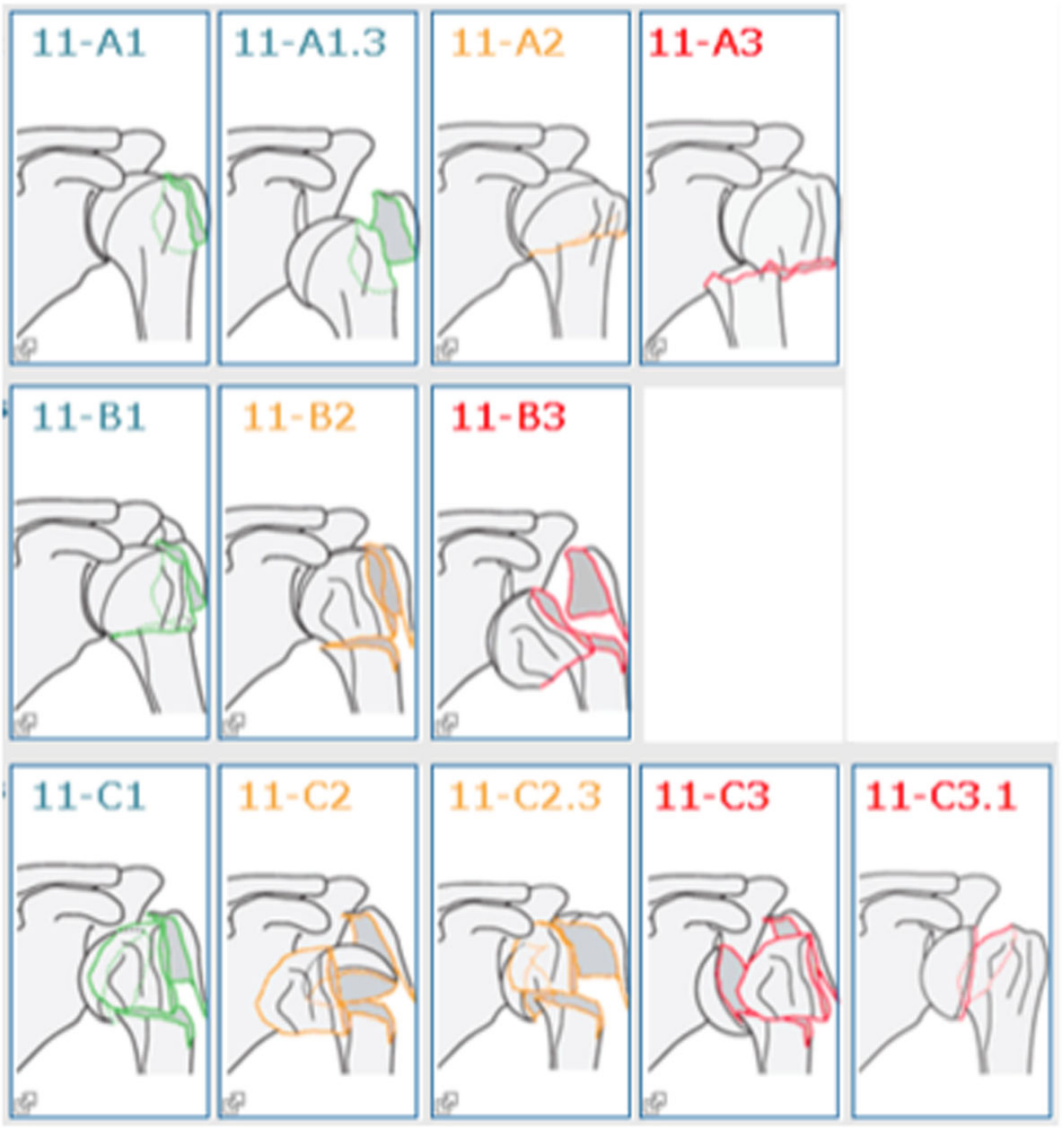
Fracture groups as presented in the SFR

Fracture classification is carried out by indicating the fracture location on the image of a skeleton (with courtesy of AO foundation), e.g. on the left proximal humerus. This image expands to display a grid with the different fracture groups and the one corresponding to the relevant fracture is chosen. For the SFR, the AO/OTA system has been used as it is a comprehensive classification system that covers most body regions [4]. Slight modifications have been made by selecting fracture subgroups from a more detailed level in order to improve the logic of the system e.g. in the SFR, for proximal humerus fractures in addition to the nine AO/OTA groups (A1-C3), there are three subgroups to enable the classification of unifocal fractures with dislocation (A1.3), head split fractures (C2.3) and pure anatomical neck fractures (C3.1), fracture types not found at group level (Fig. 1). When possible, the similarities between other frequently used classification systems, Neer, and the AO/OTA system have been highlighted to make it more “friendly” to the user [5]. Still the quality and usefulness of the data in a register such as the SFR is dependent on the accuracy of the classification of fractures. Previous studies on accuracy of the classification of tibia and ankle fractures have shown moderate to substantial accuracy [6, 7]. A study on the epidemiology of humerus fractures based on data from the SFR was recently published [8].

The aim of this study was to analyse the accuracy of the classification of humerus fractures as it is used in daily practice in the SFR. Our second aim, while analysing the subgroup of proximal humeral fractures, was to get a deeper understanding of the generally low reliability of fracture classification using concepts from “fuzzy logic” [9].

### Ethics

The study was approved by the Central Ethical Review Board, Gothenburg (ID 999–13).

## Methods

**Fig. 2:**
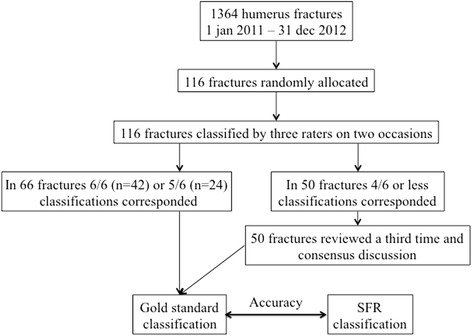
Flow chart of how the study was conducted

In January 2014, 116 humerus fractures (among them 90 proximal humeral fractures) were selected by computerised randomisation from the 1772 humerus fractures (1374 proximal humeral fractures) registered in 2011 and 2012 in the Swedish Fracture Register at Sahlgrenska University Hospital (Fig. 2). One of the authors (SS) acquired all the radiological investigations that were available for each patient at the time of registration in the SFR. The patients with proximal humeral fractures were all investigated with a standard radiographic series of three planes, i.e. anterior-posterior, lateral, and axial view except in 23 patients where the axial view was not done. 12 of the 116 patients were investigated with a CT-scan – 7/90 of the proximal humeral fractures, 5/11 of the distal humeral fractures and none of the diaphyseal humeral fractures. On two occasions, 6 weeks apart, all the images were presented to the three observers (two senior trauma surgeons and one trauma/shoulder surgeon) for independent assessment and fracture classification. No information about the patients’ age, gender or subsequent treatment was given. On all occasions, handouts and the drawings used in the SFR were available, with a detailed description of the classification system. In this way all fractures were classified six times. In cases where five or six out of six classifications corresponded this classification was considered the true classification of the fracture i.e. the gold standard classification. Once the compilation of the data was completed, a third classification session took place for the fractures for which complete agreement had not been reached. Remaining disagreement was resolved after an open discussion between the three observers and the final result was named the gold standard. The original classification in the SFR was then compared with the gold standard classification.

For the proximal humerus fractures the classification system was further analysed. The 12 fracture groups can be defined by eight Boolean questions (yes/no) (Table 1) and one question to determine the segment, similar to the work by Shrader et al. [10]. To understand the grounds for classification disagreement, the possible relationship between fracture groups was analysed. Fracture groups or subgroups separated by only one question were regarded as “related”, with the exception of “glenohumeral dislocation”. Fractures differing in two or more questions are regarded as being unrelated. “Related” fractures differ by only one question and one could be mistaken for the other if the defining fracture line is vague (e.g. whether or not there is a fracture of the greater tuberosity, or whether or not the fracture is impacted/stable).

**Table 1:** Question answered yes/no (1/0) defining the fracture groups of proximal humerus fractures. The “0”s have been omitted for clarity

	A1	A1.3	A2	A3	B1	B2	B3	C1	C2	C2.3	C3	C3.1
Two-part extra-articular	1	1	1	1								
Tuberosity only	1											
Three-part, extra-articular – bifocal					1	1	1					
Four-part, articular								1	1	1	1	
Glenohumeral dislocation		1					1				1	
Metaphyseal impaction/stable			1		1			1				
Anatomical neck only												1
Head split									1			
Proximal segment (inside the square)	1	1	1	1	1	1	1	1	1	1	1	1

### Statistical analysis

Sample size calculations were made based on kappa statistics from previous studies [1, 2, 10–14]. Based on the kappa values in these previous studies an approximate kappa value of 0.5 was expected. In order to achieve a 95% confidence interval that did not span more than one category on the scale defined by Landis and Koch, a relative error of 20% corresponding to kappa ±0.1 was accepted [15]. Intra- and inter-observer agreement analysis was performed, calculating the kappa coefficients and confidence intervals using SAS software. For the fracture groups that were regarded as “related” fractures, an intermediate fracture group was created, e.g. for the fractures assessed as “A2” in one instance and as “B1” in another, the intermediate group “A2-B1” was constructed to classify these fractures (Table 2). In this way, ten intermediate groups were constructed.

**Table 2:** Intermediate fracture groups, constructed on the boundary between “related” groups, i.e. groups separated by only one of the defining questions listed in Table 1

A1-B1	
A1.3-B3	
A2-A3	
A2-B1	
B1-B2	
B1-C1	
B2-C2	
B2-C2.3	
C1-C2	
C2-C2.3	

## Results

Age and gender distribution for all humerus fractures is presented in Table 3. Accuracy, defined as agreement between the classification in the SFR and gold standard classification, for all humerus fractures was kappa 0.57 for AO/OTA group (four signs) and 0.66 for AO/OTA type (three signs) (Table 4).

**Table 3:** Distribution of patients according to age, gender and fracture segment (proximal humerus, diaphyseal humerus and distal humerus) as defined by gold standard classification

	Women	Men	Total
Median age (range)	71 (19–102) *n* = 81	50 (16–92) *n* = 35	67,5 (16–102) *n* = 116
Median age (range) among proximal humeral fractures (AO/OTA 11XX)	71 (19–102) *n* = 67	58,5 (17–92) *n* = 22	69 (17–102) *n* = 89
Median age (range) among diaphyseal humeral fractures (AO/OTA 12XX)	82 (30–90) *n* = 8	34,5 (18–72) *n* = 8	54,5 (18–90) *n* = 16
Median age (range) among distal humeral fractures (AO/OTA 13XX)	67 (48–93) *n* = 6	37 (16–82) *n* = 5	60 (16–93) *n* = 11

**Table 4:** Percentage of agreement (PA) and Cohen’s Kappa coefficient with 95% confidence interval for accuracy, defined as SFR classification compared with gold standard classification (GS) for all humerus fractures

	Accuracy	
	SFR vs GS	
	PA	Kappa (95% CI)
AO/OTA group (4 signs)	61%	0.57 (0.47-0.67)
AO/OTA type (3 signs)	75%	0.66 (0.55-0.76)

AO/OTA group – 4 signs refer to a full AO/OTA classification with 4 signs e.g. 11A1. AO/OTA type – 3 signs refer to a simplified AO/OTA classification with 3 signs only e.g. 11A

### Proximal humerus fractures

The distribution of proximal humerus fractures between the fracture groups as determined by the gold standard classification is similar to the distribution described by Court-Brown et al. for an Edinburgh population (Table 5) [16].

**Table 5:** Comparison of the relative distribution (%) of proximal humeral fractures by AO/OTA groups in the SFR assessed by the gold standard and the Edinburgh population [16]

AO/OTA group	SFR	Court-Brown et al.
A1	10,4	14
A1.3	4.6	5
A2	24.2	27
A3	18.4	20
B1	23.0	19
B2	15.0	7
B3	1.2	0.6
C1	1.2	1.1
C2	0	2.4
C3	1.2	2.3

Full intra-observer agreement for the three observers was seen in 71, 58 and 56 respectively of the 90 cases of proximal humerus fractures. The kappa values of the intra-observer analysis are given in Table 6. When inter-observer agreement between the SFR and the gold standard classification was tested, complete agreement was seen in 57 of the 90 cases.

**Table 6:** Intra-observer kappa values with upper and lower confidence interval (CI) for proximal humerus fractures, calculated with or without taking “related” fractures into account (intermediate groups)

	Without intermediate groups		With intermediate groups	
	Mean kappa value	95% CI	Mean kappa value	95% CI
Rater 1	0.752	0.652 – 0.853	0.974	0.939 – 1.000
Rater 2	0.599	0.481 – 0.717	0.910	0.846 – 0.974
Rater 3	0.593	0.478 – 0.707	0.914	0.853 – 0.975

If the ten intermediate groups were included, complete intra-observer agreement was seen in 89, 86 and 81 of the 90 cases. When comparing the SFR with the gold standard classification with the ten intermediate groups included, 27 fractures could be classified as belonging to one of these. As a result, complete agreement was seen between the SFR and the gold standard classification in 84 of the 90 tested cases. The kappa values are given in Table 7.

**Table 7:** Inter-observer kappa values with upper and lower confidence interval (CI) for proximal humerus fractures, calculated with or without taking “related” fractures into account (intermediate groups)

	Without intermediate groups		With intermediate groups	
	Mean kappa value	95% CI	Mean kappa value	95% CI
Gold vs SFR	0.577	0.457 – 0.697	0.912	0.850 – 0.974

## Discussion

This paper has two aims: to analyse the reliability of fracture classification as it is used in daily practice and to understand the limitations of fracture classification validity. When the accuracy of classification in the SFR, as carried out by junior doctors at an emergency department, was tested against the senior consensus group, the kappa value was in the range of previous studies (0.57) [1, 2, 10–14]. Although this result corresponds to moderate agreement, according to the criteria formulated by Landis and Koch, we suggest that this result is as good as could be expected, considering that the registering doctors were under the time pressure of the emergency ward and that they were not specifically trained for the task and perhaps not even completely motivated [15]. The results are in accordance with similar studies on tibia and ankle fractures in the SFR [6, 7]. The fact that there was a considerable resemblance between the epidemiology of the randomly acquired cases and that of the incidence analysis from Edinburgh supports our notion that the classification as used in the SFR has good validity and that the selected cases are representative of the normal fracture population [16]. Conversely, it also supports the data in the report by Court-Brown et al., although only one person carried out their classification, without intra-observer error being tested.

Systems for classifying proximal humeral fractures have been developed, based on the four segments of epiphyseal union as defined by Codman and subsequently modified by Neer [5]. Another modification has been proposed by Hertel [17, 18]. These systems comprise up to 16 different fracture groups. A slightly different system has been introduced by AO/OTA, based on the generalised system for classifying fractures in the proximal or distal segment of long bones but adapted for the shoulder, which is used in the SFR.

It has been suggested that the poor inter- and intra-observer agreement that has been observed using any classification system is due to the surgeons’ inability to accurately interpret the fracture. Despite the use of CT imaging in some studies, it has not been shown to improve the results uniformly [2, 11–13, 19]. The kappa values obtained from these studies improve only slightly or not at all, when the classification systems were simplified or reduced to two options, displaced or undisplaced [1, 14, 20]. However, it has been suggested in one study that the use of CT-based stereo visualisation may substantially improve classification reliability, which has also been seen in tibial plateau fractures [21, 22].

We believe that, regardless of imaging methods or classification protocol, there is always going to be some degree of disagreement between observers. Any classification system basically attempts to divide a continuum of all the different fracture types into discreet groups. Regardless of system or imaging methods, there are always going to be boundary problems for fractures with the characteristics of two or more fracture groups. Boundaries between fracture groups could be defined by Boolean questions to be answered “yes” or “no” and a list of these questions could be used as an analytical pathway [10]. Fractures on either side of a boundary, separated by only one question, could be regarded as related and deviating assessments are possible depending on how subtle the differentiating feature is.

This is similar to the reasoning behind “fuzzy sets”. A “fuzzy set” is “a class with a continuum of grades of membership” [9]. In recent years, mathematics and the understanding of sets with imprecise properties have been developed and have found applications in numerous fields, such as the automated determination of vertebral column disorders and weed classification for precision herbicide application [23–25]. When dealing with the imprecise nature of fracture classification, it should also be pointed out that there is no “perfect truth” – it is instead a question of weighing “expert” opinions.

Rather than describing “grades of membership” for individual fractures that did not perfectly fit the definition of one fracture group alone, we introduced intermediate groups where appropriate, to comprise fractures with the features of two adjoining groups. Having introduced the intermediate groups, the kappa value rose from 0.0577 to 0.912, when the SFR was compared with the “gold standard”, and from 0.593–0.752 to 0.910–0.974 for the intra-observer comparisons. Another way to describe this is that in most of the cases where there is disagreement, the disagreement is between “related fracture groups”. The unadjusted kappa values correspond to what has previously been published. On the other hand the adjusted kappa values are exceedingly high and have to be interpreted with caution. The high, adjusted kappa values may represent a near upper limit of agreement when the boundary problem is taken into account. Two surgeons with a divergent view of slight details that may change the classification are still likely to perceive the fracture in the same way, as long as the fracture groups are “related”. This may explain the higher agreement for treatment recommendations than for the fracture classification [26].

### Strengths and limitations of the present study

Although sample size calculations were made the 116 randomly allocated humerus fractures included only 16 diaphyseal and 11 distal humerus fractures. However the statistical analysis was done on all humerus fractures and proximal humerus fractures respectively and the low numbers of diaphyseal and distal humerus fractures therefor should not affect the reliability of results. The current study has the same design and similar results as the previous studies on the validity of tibia fracture and malleolar fracture classification in the SFR [6, 7]. With the AO/OTA classification system classifying the correct segment could pose a problem, e.g. whether the fracture belongs to segment 1 (proximal) or segment 2 (diaphysis). In the current study there was disagreement regarding to what segment a fracture should be assigned in seven cases (in three cases between gold standard and the SFR and in four cases within the consensus group). We therefor find it important to study the whole of humerus. We also think it is important that the studies on validity of fracture classification in the SFR are of the same design. The inter-observer variation that was tested used data extracted from the SFR, against the “gold standard”. We have therefore tested the accuracy of the classification system as used in real life, by doctors not specifically trained and not with the mind-set of a test situation. The introduction of “intermediate groups” has several limitations. The purpose of this is not to introduce a new classification system to be used in clinical practice. The purpose is merely to introduce a new way of analysing agreement in fracture classification that shows that most disagreements are between the “related fracture groups”. The test was carried out on a fairly large number (*n* = 116) of randomly acquired fractures with seemingly normal distribution and we believe that we have thus adhered to most of the quality criteria set by Audigé et al. [27]. We believe that the normative “gold standard” that we have attempted to establish is fairly close to the “truth”, based as it is on multiple classification rounds followed by analytical discussions. By comparison, the classification presented in the study of the epidemiology of proximal humeral fractures [16], was carried out by only one person and no intra-observer variations were tested.

## Conclusions

The classification of humerus fractures in the Swedish Fracture Register is as accurate as in previous studies. We also believe that we offer a new way of interpreting the low kappa values of fracture classification by attempting to analyse the borderline problems that exist with any classification.

## Abbreviations

AO: Arbeitsgemeinschaft für osteosynthesefragen
CT: Computed tomography
Eq5D: Euroqol 5 dimensions
ICD-10: International Classification of Diseases Tenth Revision
OTA: Orthopaedic Trauma Association
PROM: Patient Reported Outcome Measures
SFR: Swedish Fracture Register
SMFA: Short Musculoskeletal Function Assessment
